# Assessment of the Quality of Service Given by Health Care Provider about Tuberculosis in RNTCP

**DOI:** 10.4103/0970-0218.66870

**Published:** 2010-04

**Authors:** Rashmi, B Vijaykumar

**Affiliations:** Assistant Professor, Department of Community Medicine, K.S.Hegde Medical Academy, Delakatte, Mangalore - 18, India; 1 Professor, Department of Community Medicine, J.J.M.Medical College, Davangere - 2, India. E-mail: csrashmi@yahoo.com

Sir,

The directly observed therapy short-course (DOTS) is an effective and widely accepted strategy for TB control. Few countries find success in expanding DOTS coverage to enough people to meet global targets. The main constraints to achieving the global targets include lack of political commitment, insufficient and ineffective use of financial resources, neglect of human resource development, poor health system organization, poor quality and an irregular supply of anti-TB drugs, and weak communication components in TB control programs.[1] This study was conducted in Davangere of Karnataka state in India a) to assess the quality of service in the delivery of tuberculosis care, b) to evaluate client satisfaction with the government approach and c) to assess utilization of facilities by the community. Multistage sampling was done. The district was stratified into taluks. There are 6 taluks, so 6 PHC, 5 PHU and 1 CHC in this district. Totally, 12 health centers were selected. The sample constituted 12.25% of the total health centers of Davangere.[2] A ‘*rule of thumb*’ was used for the rough estimation of sample size.[3]

Quality assessment was done using the above rule where in each health centre had total service provider <50, so 30–50% of the sample among service provider was taken. One doctor in each centre wherever doctor is present and 20-30% of JHW (F) were selected.[3] There were 9 centers where the doctors were available full time, so sample was 9. We had 17 JHW (F) in 12 centres instead of 18 JHW (F). Client satisfaction was assessed with 30 clients as the sample.[3] Utilization of services was assessed using households as population. As per thumb rule, when household exceeded >100, 10% of the houses were sampled. When the household exceeded <100, >50, 20% of the households were taken and when it was <50, 30% of the households were sampled.[3] Using this method, the total number of households were found to be 478 in the district. The areas covered by JHW (F) sampled for quality of work were also taken for the survey of utilization of services.

Module 6 of Agha Khan Foundation was used for quality assessment of services. The questionnaires were modified according to local needs and was pretested before data were collected.[3] Single observer was used to avoid bias for viewing the services. Not a single client was interviewed in front of any service provider so that strict confidentiality was maintained. Client satisfaction was assessed based on time span, referred system, facilities etc.

A total of 9 out of 12 centres dealt with tuberculosis, no health worker or doctor was present in one center, patients were directly sent to DTC in one urban center, and finally one sub-centre area did not cater the required service, instead the patients were directly referred to higher centre. History taking was well done (64.82%), 89% health workers asked about cough, 100% asked about fever, and 66% asked for blood in sputum and weight loss, but only 22% tried to find out the source of infection. Examination of the patient was satisfactory. Their work was excellent in those areas where doctor was present full time. 100% of the health workers auscultated the lungs, only 22% took temperature and weighed patients, and 78% of the health workers examined the lymph nodes of patients. Referral was done and treatment was given in almost all centers (97.22%). Follow-up of cases was well done (81.48%). All centres had adequate supplies (100%). However, health education given to patients with tuberculosis was very poor (45.83%). 100% of the health workers told about lab tests and completion of treatment. Only 22% told about the spread and contagiosity of disease, 33% told about the follow-up. None told about the adverse effects of treatment and no patients were allowed to ask any question. Service provider’s knowledge was excellent and the patients’ knowledge was also good, as evident from the exit interview of the patients.

Tuberculosis care was good on the whole. But health education was average because there was no emphasis on health education in RNTCP which is reflected by the service provider, similar to the findings of the earlier study.[4] Because of excessive importance to RNTCP, both knowledge and practice about tuberculosis was good. This is unlike the results of earlier studies.[4,5] Client satisfaction was very good, except 53.33% felt that facilities and equipments were poor, because most of the centers did not have microscope and patients were referred to other centers which was not felt as appropriate. Also the clients were not happy with the time of referral as reported in an earlier study.[6] The clients were dissatisfied because they did not get proper answers about treatment and side-effects, also some of their questions were not answered. 13% were not satisfied about the duration of waiting. In an earlier study[6] utilization of services was found to be not satisfactory, but we have 100% utilization and we assume that the emphasis on RNTCP might have made it more user friendly, which is similar to the findings of a Tanzania-based study.[7]
